# Role of Autophagy in the Maintenance of Stemness in Adult Stem Cells: A Disease-Relevant Mechanism of Action

**DOI:** 10.3389/fcell.2021.715200

**Published:** 2021-08-03

**Authors:** Shanshan Chen, Wenqi Wang, Hor-Yue Tan, Yuanjun Lu, Zhiping Li, Yidi Qu, Ning Wang, Di Wang

**Affiliations:** ^1^School of Life Sciences, Jilin University, Changchun, China; ^2^Centre for Chinese Herbal Medicine Drug Development, School of Chinese Medicine, Hong Kong Baptist University, Hong Kong, China; ^3^School of Chinese Medicine, The University of Hong Kong, Hong Kong, China; ^4^Engineering Research Center of Chinese Ministry of Education for Edible and Medicinal Fungi, Jilin Agricultural University, Changchun, China

**Keywords:** autophagy, adult stem cells, self-renewal, proliferation, differentiation, cancers

## Abstract

Autophagy is an intracellular scavenging mechanism induced to eliminate damaged, denatured, or senescent macromolecular substances and organelles in the body. The regulation of autophagy plays essential roles in the processes of cellular homeostasis and senescence. Dysregulated autophagy is a common feature of several human diseases, including cancers and neurodegenerative disorders. The initiation and development of these disorders have been shown to be associated with the maintenance of disease-specific stem cell compartments. In this review, we summarize recent advances in our understanding of the role of autophagy in the maintenance of stemness. Specifically, we focus on the intersection between autophagy and adult stem cells in the initiation and progression of specific diseases. Accordingly, this review highlights the role of autophagy in stemness maintenance from the perspective of disease-associated mechanisms, which may be fundamental to our understanding of the pathogeneses of human diseases and the development of effective therapies.

## Introduction

Stem cells are a group of self-renewing cells that originate during embryogenesis and persist throughout the lifespan of the organism. These cells can be classified into two main types based on the stage of origin: embryonic stem cells (ESCs) and adult stem cells (Boya et al., 2018). ESCs are characterized by unlimited proliferation and a pluripotent state, whereas adult stem cells generally originate and reside in specific tissues and have a limited differentiation capacity (Boya et al., 2018). Under physiological conditions, stem cells largely remain quiescent and retain their stemness; however, they are able to either proliferate or differentiate to meet regenerative demands and maintain tissue homeostasis. In this way, the balance between the growth and degeneration of tissues and organs can be maintained by the dynamic status of stem cells.

Existing evidence indicates that the fate of adult stem cells is influenced by several factors, including growth factors, the cellular niche, metabolic pathways, calcium homeostasis, and autophagy.

Growth factors are essential signaling molecules that promote the proliferation and differentiation of adult stem cells. These factors interact with cell-cell contacts and cell-matrix adhesions to engineer a microenvironment that regulates the survival and fate of stem cells. For example, in random skin flaps used to treat type 1 diabetes mellitus, the release of vascular endothelial growth factor improves the ability of mesenchymal stem cells (MSCs) to repair ischemia via angiogenesis (Chehelcheraghi et al., 2019). Fibroblast growth factor-2, a mitogen, shortens the cell cycle and improves the proliferation efficiency of neural stem cells (NSCs) (Hicks and Miller, 2019). In contrast, transforming growth factor-β1 (TGF-β1) promotes cell cycle exit and inhibits the proliferation efficiency of NSCs (Hicks and Miller, 2019).

The cellular niche, also known as the microenvironment, is composed of neighboring cells and extracellular matrix and contains a reservoir of stem cells (Vazin and Schaffer, 2010). Notably, mechanical and physical cues from the niche are required for adult stem cells to maintain their potency during tissue development and regeneration (Vining and Mooney, 2017). Accordingly, the niche facilitates the essential functions of adult stem cells in maintaining tissue homeostasis and responding to foreign stimuli.

The mitochondrion is traditionally described as the powerhouse of the cell due to its fundamental role in energy production (Xu et al., 2013). However, in the tissues and organs of an adult organism, pluripotent stem cells preferentially use non-oxidative glycolysis as the major source of energy and display reduced mitochondrial mass and oxidative phosphorylation (Rehman, 2010). Consistently, hypoxia is a potent suppressor of mitochondrial oxidation and it appears to promote the stemness of adult stem cells (Rehman, 2010). Accordingly, undifferentiated stem cells are characterized by low mitochondrial oxidative phosphorylation levels. Moreover, this cell population maintains pluripotency and self-renewal by regulating mitochondrial oxidative metabolism.

Calcium (Ca^2+^) is a ubiquitous intracellular signaling molecule that regulates differentiation, proliferation, and apoptosis. As an intracellular messenger, Ca^2+^ plays a crucial role in cell signaling pathways during various stages of stem cell differentiation (Tonelli et al., 2012). For example, after being induced by increased extracellular Ca^2+^ levels, bone morphogenetic protein-2 promotes the osteogenic differentiation of human adipose-derived stem cells (Yanai et al., 2019).

Autophagy, which literally translates as “self-eating,” is a cellular process of self-digestion, by which intracellular compartments are scavenged within the cell (Chang, 2020). Thus far, three main types of autophagy have been identified in mammalian cells: microautophagy, chaperone-mediated autophagy, and macroautophagy (Scrivo et al., 2018). Macroautophagy is the prevalent form of autophagy, and is hereby referred to as “autophagy.” It is a highly conserved process for the degradation and recycling of damaged and aged proteins and organelles, which thereby prevents cellular damage.

The autophagy-mediated recycling of cellular components is a critical step in cell homeostasis and tissue remodeling during development (Chang, 2020). More than 30 autophagy-related genes (*Atgs*) have been identified by screening yeast for autophagy-absent abnormalities. These genes are also evolutionarily conserved in a diverse range of species. The proteins encoded by these genes form a series of complexes that engage in different stages of the autophagic process, including autophagy induction, autophagosome formation, and the fusion of autophagosomes and lysosomes (Wang et al., 2019). Autophagy induction is regulated by the mammalian target of rapamycin (mTOR) complex 1 (mTORC1) pathway (Wang et al., 2019). Under conditions of starvation, the inhibition of mTORC1 promotes the formation of the ULK complex, which includes Atg13, ULK (uncoordinated-51 like kinase) 1, FIP200 (an interacting protein of the focal adhesion kinase family), and Atg101, thus leading to the activation of autophagy. Studies have also shown that 5′-AMP-activated protein kinase (AMPK) is involved in autophagy induction by suppressing mTOR (Liu L. et al., 2017). The class III phosphatidylinositol 3-kinase (PI3K) complex I translocates to the endoplasmic reticulum (ER) after the formation of the ULK complex. The ULK and PI3K complexes then promote the nucleation and assembly of the isolation membrane (IM) to form the precursor of the autophagosome. Atg9 is also recruited by the ULK complex to the IM to carry membrane components used for IM expansion. Furthermore, two ubiquitin-like conjugation systems, the Atg12 conjugation system (Atg12-Atg5-Atg16) and the microtubule-associated protein light chain 3 (LC3) system, facilitate IM expansion and the final formation of the autophagosome (Geng and Klionsky, 2008). The mature autophagosome then fuses with lysosomes or endosomes to form an autolysosome. The contents of the autolysosome are degraded and recycled to the cytoplasm for further utilization (Wang et al., 2019).

**FIGURE 1:**
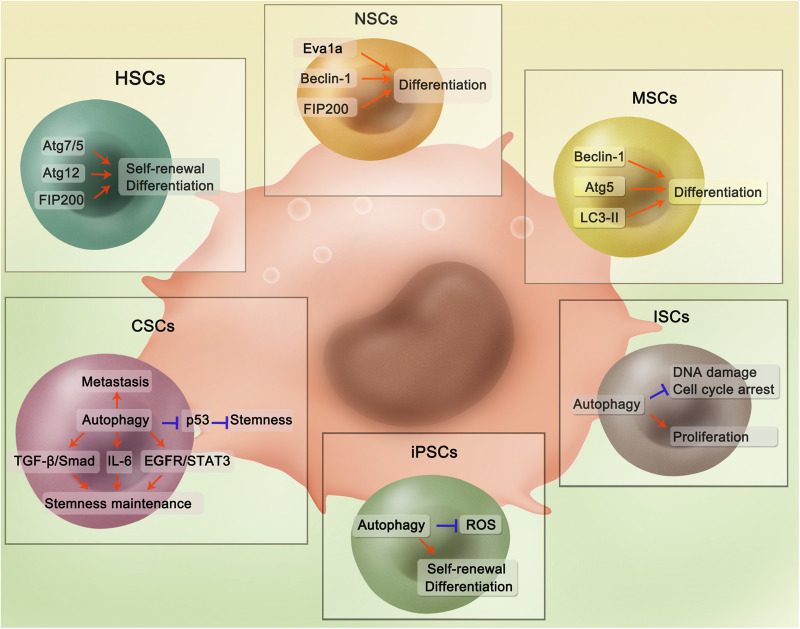
Regulatory mechanisms of autophagy in different adult stem cells. In HSCs, Atg7/5, Atg12, and FIP200 are able to maintain stemness and promote differentiation. Autophagy-related proteins Eva1a, Beclin-1, and FIP200 could promote NSCs differentiation through activating autophagy. In MSCs, Beclin-1, Atg5, and LC3-II could contribute to differentiation. For ISCs, Autophagy could reduce DNA damage and cell cycle arrest with ISCs proliferation also promoted. For iPSCs, autophagy is implicated in the inhibition of ROS-induced injury and promotion of self-renewal and differentiation. Autophagy plays an important role in CSCs through several pathways. Specifically, in the hepatic cancer stem cells, mitophagy could suppress p53 which hampers stemness maintenance. While in the breast cancer, autophagy regulates self-renewal of several breast cancer stem cells through different pathways including TGF-β/Smad, IL-6 secretion, and EGFR/STAT3. Besides, autophagy is involved in the tumor metastasis, which is evidenced in various cancers.

In recent years, an increasing number of studies have shown that autophagy plays an essential role in the maintenance of homeostasis and stemness in both ESCs and adult stem cells under physiological conditions, as well as in the initiation and progression of human diseases (Garcia-Prat et al., 2016; Zuo et al., 2019). In ESCs, autophagy activation is important for the maintenance of their stemness (Wang et al., 2019). For example, it has been shown that the pluripotency of ESCs is inhibited by ULK1 deficiency (Gong et al., 2018). Ectopic P-granules autophagy protein 5 homolog (EPG5) is a eukaryotic-specific autophagy regulator that mediates the fusion of autophagosomes and lysosomes. Mechanistically, the non-classical K63-linked ubiquitin chain of EPG5 is removed by the ubiquitin-specific peptidase USP8, an enzyme that catalyzes deubiquitination. Once EPG5 is deubiquitinated, the interaction between EPG5 and LC3 is enhanced, which results in increased autophagy flux, thus promoting the maintenance of ECS stemness (Gu et al., 2019). However, compared to the role of autophagy in maintaining the stemness of ESCs, its role in maintaining the stemness of adult stem cells is more complicated due to different types of adult stem cells, as discussed in detail in the follow section. The involvement of autophagy-related genes and proteins in the proliferation and differentiation of stem cells, as well as the maintenance of their stemness, has been intensively studied in several types of adult stem cells, including hematopoietic stem cells (HSCs), NSCs, MSCs, intestinal stem cells (ISCs), induced pluripotent stem cells (iPSCs), and cancer stem cells (CSCs) (Figure 1). More importantly, impaired autophagy has been shown to contribute to the pathogenesis of various diseases, such as cancer (Zhu et al., 2013; Yao et al., 2020), neurodegenerative disorders (Park et al., 2014), and responses to viral infections (Tiwari et al., 2020). Therefore, in this review, we will also discuss the roles that autophagy plays in stem cells from the point of view of stem cell-related diseases, to facilitate improved utilization of autophagy-related targets for clinical therapies.

## The Physiological Roles of Autophagy in Different Adult Stem Cells and Related Diseases

### Physiological Role of Autophagy in HSCs

Hematopoietic stem cells are the primary source of all blood cells, and they maintain blood homeostasis by producing myeloid- and lymphoid-precursor cells (Birbrair and Frenette, 2016). Myeloid-precursor cells differentiate further to produce the majority of white blood cells, while lymphoid precursor cells differentiate into lymphocytes. HSCs are distributed ubiquitously in the extremely hypoxic and specialized bone marrow niche. They contain relatively few mitochondria to keep mitochondrial oxidative phosphorylation quiescent and maintain their self-renewal ability. Based on the duration of their stemness, HSCs can be divided into long-term HSCs (at least 6 months) and short-term HSCs (up to 3 months) (Zon, 2008).

The self-renewal of HSCs is regulated via various pathways. For example, in long-term HSCs, pyruvate dehydrogenase kinase activation induced by hypoxia inducible factor (HIF-1α) inhibits pyruvate dehydrogenase, which promotes the maintenance of glycolytic flow and the inhibition of mitochondrial oxidative phosphorylation, thus maintaining cell cycle quiescence in HSCs (Takubo et al., 2013). Apart from anaerobic glycolysis mentioned above, autophagy, especially mitophagy (a specific selective term for autophagy related to mitochondrial removal), also plays an important role in HSC self-renewal. Damaged and redundant mitochondria are eliminated and recycled via mitophagy to sustain the stemness of HSCs (Murakami et al., 2021). Impaired mitophagy, presenting as an increase in mitochondrial respiration and subsequent oxidative stress, causes an increase in reactive oxygen species (ROS) production and limits self-renewal potential, resulting in the transformation of long-term HSCs to short-term HSCs (Koschade and Brandts, 2020). Although short-term HSCs cannot maintain their stemness for as long as long-term HSCs, they generate hematopoietic progenitor cells, which may further differentiate into blood cells (Koschade and Brandts, 2020). In colony-forming cellular assays, when autophagosome formation is blocked with 3-methyladenine (3-MA), the number of HSCs decreases and self-renewal capacity is inhibited, which is similar to the results when Atg5 expression is blocked with an *Atg5*-specific shRNA (Salemi et al., 2012). In the *Atg12*^*flox/flox*^:*Mx1-Cre* conditional knockout (*Atg12*^*cKO*^) mouse model, enhanced mitochondrial production and accelerated oxidative phosphorylation increased the entry of HSCs into the cell cycle, which disrupted their quiescence (Ho et al., 2017). Liu et al. reported that augmented mitochondrial mass and ROS levels in FIP200-deficient fetal HSCs prevent their stemness maintenance (Liu et al., 2010). Consequently, these results indicate that autophagy contributes to the regulation of HSC stemness.

In addition to its role in stemness maintenance, exogenous TGF-β1 has been shown to increase the rate of differentiation of HSCs to red blood cells by stimulating mitophagy (Kuhikar et al., 2020). FIP200 is involved in the differentiation of HSCs to mature red blood cells, and therefore, the depletion of FIP200 inhibits erythropoiesis (Zhang et al., 2015). Similarly, the conditional deletion of Atg7 in HSCs leads to the accumulation of mitochondria and superoxide, resulting in a failure to differentiate into myeloid- and lymphoid-precursor cells (Mortensen et al., 2011).

### Disease-Relevant Mechanism of Autophagy in HSCs

A defect in the balance between the self-renewal and differentiation of HSCs leads to various diseases related to the hematopoiesis system. Hematopoietic cells convert into leukemia cells at the stem cell (leukemia stem cells [LSCs]) or progenitor cell stage (Koschade and Brandts, 2020). As a double-edged sword, autophagy may delay or contribute to the initiation and progression of HSC-related diseases. Here, we will discuss three major diseases related to HSCs, namely, acute myeloid leukemia (AML), chronic myeloid leukemia (CML), and B-cell acute lymphoblastic leukemia (B-ALL), and focus on the role of autophagy in these diseases.

Atg5- or Atg7-deficient hematopoietic stem and progenitor cells exhibit defective autophagy, which leads to bone marrow hyperplasia, dysregulated glycolytic metabolism, and an increased susceptibility to AML (Watson et al., 2015). CD147 is a transmembrane protein that performs various functions in cancers, including AML. Studies have found that CD147 levels increase in cancer cells, resulting in tumor progression and chemotherapeutic resistance. Isabella et al. found that AC-73, an inhibitor of CD147, decreases leukemia cell proliferation through the inhibition of the extracellular-signal-regulated kinase (ERK)/signal transducer and activator of transcription 3 (STAT3) pathway and the activation of autophagy. They also reported that CD147 is co-expressed in leukemia cells with CD371, a surface marker of LSCs, suggesting that AC-73-induced autophagy induction in LSCs may be a promising strategy for AML treatment (Spinello et al., 2019). Accordingly, these results demonstrate that autophagy activation helps delay the progression of AML. However, autophagy can also be instrumental in LSCs, which aggravate the disease. AMPK/FIS1-mediated intrinsic mitophagy plays an important role in clearing stressed mitochondria, thus maintaining the self-renewal capacity of LSCs (Pei et al., 2018). Furthermore, autophagy helps form a drug-resistance mechanism to protect LSCs against apoptosis. The effect of the bromodomain and extraterminal domain inhibitor JQ1 on AML is limited, partly because AMPK-ULK1-mediated autophagy is activated to decrease LSC apoptosis (Jang et al., 2017). Therefore, even though autophagy plays a dual role in AML development, targeting autophagy remains a promising therapeutic strategy for AML.

Chronic myeloid leukemia is caused by the malignant transformation of HSCs, and its pathogenesis involves BCR/ABL tyrosine kinase (Bellodi et al., 2009). Imatinib is a tyrosine kinase that has been used for CML therapy; however, the benefits are limited due to the development of drug resistance. Researchers have previously found that imatinib induces protective autophagy in LSCs (Bellodi et al., 2009). Accordingly, combined treatment with an autophagy inhibitor and a tyrosine kinase inhibitor, such as imatinib, was found to almost completely eliminate LSCs. However, a recent study confirmed that combined treatment with a histone deacetylase inhibitor and imatinib increases the apoptosis of LSCs, partly due to autophagy activation, as knockout of the autophagy-related gene *BECN1*, which encodes Beclin-1 (inducer of autophagosome formation), was found to inhibit LSC apoptosis (Lernoux et al., 2020). The effect of this type of combined therapy on LSCs requires further exploration. Collectively, these findings provide robust evidence for the need to investigate CML from the view of autophagy and LSCs.

B-cell acute lymphoblastic leukemia mostly occurs in children and is caused by the malignant transformation of B cells (Delahaye et al., 2021). In patients with B-ALL, LSCs express the phenotypic surface markers CD34 and CD19. Experimentally, treatment with bafilomycin A1 (BafA1), an inhibitor of the fusion of autophagosomes and lysosomes, leads to a significant reduction in the number of CD34^+^CD19^+^ LSCs in the G0 phase of the cell cycle (Xu et al., 2020). In other words, BafA1 induces quiescent LSCs to enter the cell cycle, while retaining normal HSCs. Autophagy inactivation eventually leads to an impaired self-renewal capacity of LSCs in primary B-ALL, which is followed by apoptosis. Nevertheless, rapamycin-mediated autophagy activation promotes the survival of animals with B-ALL via the partial restoration of HSCs and cell-cycle arrest of ALL cells (Yuan et al., 2015). Therefore, both the suppression and induction of autophagy may be instrumental in B-ALL treatment. This treatment strategy should be considered and studied extensively.

### Physiological Role of Autophagy in NSCs

Neural stem cells reside in the embryonic nervous system and certain parts of the adult brain, where they generate a diverse range of new neurons to maintain homeostasis and repair damage. NSCs are mainly confined to two regions in adulthood: the subventricular zone (Yazdankhah et al., 2014) and the subgranular zone (Obernier and Alvarez-Buylla, 2019). NSCs generate a large number of neurons and glial cells during brain development, and in the subventricular and subgranular zones, they have lifelong adult neurogenesis ability, which is of great significance for learning, memory, and damage repair. Basal autophagy plays a pivotal role in brain growth, synaptic plasticity, neurodegenerative disease development, and neoplasia via its protective effects, which involve the elimination of damaged organelles and proteins. Accordingly, basal autophagy is implicated in the proliferation and differentiation of NSCs, and it also regulates the survival or death of these cells under stressful conditions. On one hand, autophagy maintains NSC homeostasis through several pathways. On the other hand, chronic restraint stress activates the autophagic cell death pathway, by which NSCs undergo programmed death (Jung et al., 2020).

Neural stem cell proliferation and differentiation are pivotal components of self-renewal and damage repair processes within the nervous system. The autophagy-related gene *EVA1A* (Eva-1 homolog A) has been shown to regulate the self-renewal and differentiation of NSCs. *In vitro* and *in vivo* studies have shown that when Eva1a is depleted, the PI3K-AKT axis is activated before mTOR activation, to inhibit autophagy, leading to embryonic neurogenic defects (Li M. et al., 2016). This study also showed that embryonic neurogenic defects caused by Eva1a depletion were recovered after adding methylpyruvate to the culture during NSC differentiation. Thus, we speculate that autophagy plays an energy-providing role during NSC differentiation. Further, Beclin-1 knockout or 3-MA treatment leads to the inhibition of autophagy and a significant suppression of NSC differentiation (Yazdankhah et al., 2014). Similarly, the conditional knockout of FIP200 in mice leads to a decrease in the number of adult NSCs, the disruption of neuronal differentiation, and the eventual disappearance of the NSC pool (Wang et al., 2013). FIP200 also regulates NSC differentiation via a novel non-cell autonomous mechanism. FIP200-deficient NSCs in the subventricular zone have increased expression levels of Ccl5 and Cxcl10, both of which are able to recruit microglia of the M1 phenotype to infiltrate this zone. Activated microglia can then indirectly inhibit the differentiation of FIP200-null NSCs (Wang et al., 2017). Other studies have also found that FIP200 activates mTOR, thus facilitating cell growth by either interfering with the formation of tuberous sclerosis complex protein (TSC)1 and TSC2 (Gan et al., 2005) or by degrading TSC1 via the ubiquitin-proteasomal pathway (Chano et al., 2006), in mouse embryonic fibroblasts and neuromuscular cells, respectively. TSC1 and TSC2 (or hamartin and tuberin, respectively) are both tumor suppressor proteins that negatively regulate mTOR. In these two studies, the finding that FIP200 regulates mTOR to promote cell growth is contradictory to previous findings that mTOR is a negative regulator of autophagy activation, which promotes cell growth and differentiation (Li M. et al., 2016). It may be interesting to explore how FIP200 regulates mTOR in NSCs and whether FIP200-mediated autophagy is involved in the control of cell growth.

### Disease-Relevant Mechanism of Autophagy in NSCs

Much attention has been paid to the relationship between NSCs and autophagy during viral infections. Zika virus (ZIKV), a flavivirus, mainly infects NSCs of the growing fetus, causing brain defects, including microcephaly (Chiramel and Best, 2018). Autophagy plays a dual role in NSCs infected by ZIKV. Virophagy, a selective form of autophagy, is used by NSCs to sequester viral components in an effort to defend against ZIKV infection (Tiwari et al., 2020). Autophagy in NSCs can also be used by ZIKV to promote its own replication in host cells by inhibiting the AKT-mTOR pathway through its non-structural proteins, NS4A and NS4B (Liang Q. et al., 2016).

Autophagy failure is a known characteristic of Alzheimer’s disease (AD) (Nixon and Yang, 2011). Such a failure is caused by presenilin 1 deficiency, as a consequence of dysregulated ERK/CREB signaling, as evidenced by a reduction in the number of autophagosomes and autophagy-related mRNA and protein levels in presenilin 1-deficient NSCs (Chong et al., 2018). Studies have confirmed that autophagy activation can ameliorate AD. Specifically, the combination of berberine and curcumin extracted from herbal medicine effectively attenuates inflammation and oxidative stress in AD via autophagy induction through the AMPK pathway (Lin et al., 2020). Another study showed that the AMPK pathway activated by resveratrol is instrumental for NSCs to ameliorate inflammation and oxidative stress caused by amyloid-beta deposition. Although the authors of this study did not mention the involvement of autophagy in inhibiting inflammation and oxidative stress in NSCs, other studies have shown that AMPK-activated autophagy helps clear amyloid-beta deposits (Rahman et al., 2021; Wani et al., 2021), which have a strong relationship with neuroinflammation and oxidative stress (Moore and O’Banion, 2002). In conclusion, these results make it reasonable to speculate that AMPK-related autophagy in NSCs is a promising target for AD treatment.

Furthermore, based on the positive regulation of mTOR by FIP200 during cell growth (Chano et al., 2006), it has also been found that in differentiated neurons, FIP200 dysfunction leads to neurite atrophy and apoptosis and eventually exacerbates AD. These effects also occur through mTOR repression (Chano et al., 2007). NSCs have the ability to differentiate into new neurons, while FIP200 engages in regulating cell growth. It may be interesting to explore the relationship between FIP200 and mTOR using NSCs as a model, to facilitate the development of novel AD treatments.

### Physiological Role of Autophagy in MSCs

The pluripotency of MSCs was first identified using cells from murine bone marrow (Friedenstein et al., 1968). MSCs can be isolated from various tissues, including bone marrow, umbilical cord, adipose tissue, liver, and pancreas, and they exhibit varying degrees of differentiation and proliferation potential.

Autophagy regulates MSC stemness and differentiation under several conditions. Autophagy may be activated in response to extreme conditions, such as hyperglycemia, senescence, increased ROS levels, or hypoxia. In this context, autophagy may be protective or destructive for MSCs (Sbrana et al., 2016). The specialized differentiation of MSCs requires autophagy. For example, upon activation by cAMP, the ERK1/2-Beclin-1 signaling pathway induces autophagy in MSCs, thus inhibiting the proliferation of MSCs and also contributing to their differentiation into nerve cells (Ugland et al., 2011). Irisin is produced after exercise and is involved in the transformation of white adipose tissue to brown adipose tissue, thus regulating energy consumption. A recent study found that irisin promotes the osteogenic differentiation of bone marrow mesenchymal stem cells (BMSCs) by activating autophagy, as evidenced by increased levels of LC3II and Atg5 (Chen et al., 2020). ROS accumulation and radiation-induced DNA damage induce a loss of stemness in autophagy-deficient MSCs, suggesting that autophagy protects MSCs against oxidative damage, thus maintaining their stemness (Hou et al., 2013). Interestingly, another study found that under hypoxic conditions, autophagy activated by the AMPK/mTOR pathway leads to MSC apoptosis (Zhang et al., 2016).

### Disease-Relevant Mechanism of Autophagy Concerning MSCs

Due to their pluripotency, the potent regenerative properties of MSCs have attracted considerable attention in research on human disease therapies. In this review, we mainly focus on the roles of autophagy in recipient cells after MSC transplantation. Specifically, Parkinson’s disease, myocardial infarction, myocardial ischemia/reperfusion (I/R) injury, and cerebral I/R injury will be discussed in detail in this section.

In Parkinson’s disease, abnormal proteins produced by mistranslation are not properly processed, causing aggregates to precipitate. The accumulation of α-synuclein, which is toxic to neurons and ultimately causes neuronal death, is commonly seen. In the 1-methyl-4-phenyl-1,2,3,6-tetrahydropyridine-treated animal model of Parkinson’s disease, levels of α-synuclein in dopaminergic neurons have been shown to decrease after MSC administration, partly due to autophagy induction (Park et al., 2014). Specifically, MSCs release certain small molecules that activate the PI3K/AKT pathway in neurons, which upregulates autophagy-related proteins, such as Beclin-1, leading to autophagosome formation and the clearance of α-synuclein (Shin and Lee, 2020). Another study found that MSC administration promotes the fusion of autophagosomes and lysosomes (Park et al., 2014). Similarly, MSC treatment induces the clearance of amyloid-beta in AD, along with the promotion of autophagosome formation and fusion (Shin et al., 2014), which may also be a promising treatment strategy for other neurodegenerative diseases. However, further studies should also be conducted to explore the exact mechanism of MSC treatment in these diseases.

In myocardial infarction, MSC treatment also regulates autophagy. The transplanted MSCs release apoptotic bodies, which are engulfed by recipient endothelial cells. Apoptotic bodies increase the expression of transcription factor EB in recipient cells. The induction of the autophagy transcription factor EB then promotes angiogenesis and the recovery of cardiac function (Liu et al., 2020).

Myocardial ischemia is defined as a lack of blood flow to cardiac tissue, which causes an oxygen imbalance, with subsequent cardiac dysfunction and myocardial tissue damage. The effective and timely restoration of blood perfusion can reduce the associated myocardial injury and necrosis, but it may cause further tissue damage. BMSCs relieve ischemic myocardial reperfusion injury via two mechanisms (Wang and Li, 2007). First, these cells can differentiate into myocardial and vascular cells. Second, BMSCs secrete various growth factors, cytokines, and exosomes and thus mediate endogenous regeneration, particularly angiogenesis, via paracrine activation of resident cardiac stem cells and other stem cells.

In the case of myocardial ischemia and associated hypoxia, the mTOR pathway is involved in autophagy regulation in BMSCs. mTOR is negatively regulated by AMPK. Hypoxia enhances AMPK/mTOR signaling pathway activity (Liu L. et al., 2017), and BMSCs play a potential role in inhibiting myocardial apoptosis via the induction of myocardial autophagy under conditions of hypoxic stress. During myocardial ischemia and hypoxia, intracellular ATP levels significantly decrease in cardiac myocytes, leading to an increased AMP/ATP ratio (Matsui et al., 2008). Activated AMPK then phosphorylates TSC2 and thus inhibits mTOR activation (Wang et al., 2018). This signaling cascade mainly suppresses protein synthesis and induces autophagy, which enables the myocardium to adapt to the hypoxic environment. When myocardial ischemia occurs, autophagy is inhibited. BMSCs promote the activation of AMPK in cardiomyocytes to block the mTOR pathway, reactivate autophagy, and inhibit apoptosis. At this time, autophagy has a protective effect on ischemic myocardial cells (Liu L. et al., 2017). Therefore, regulation of the AMPK/mTOR signaling pathway may be the mechanism of action underlying the effects of BMSCs in the treatment of myocardial I/R injury.

However, autophagy activation exacerbates cerebral I/R injury. In a rat model of cerebral I/R injury (middle cerebral artery occlusion), the intravenous transplantation of BMSCs leads to increased p-AKT and p-mTOR levels, but decreased LC3 and Beclin-1 levels, which facilitates behavioral improvements, reduced cerebral infarction volume, and decreased neuronal apoptosis (He et al., 2019). In summary, activation of the PI3K/AKT/mTOR signaling pathway protects the brain from cerebral I/R injury by suppressing autophagy.

### Physiological Role of Autophagy in ISCs

In mammals, the intestinal lumen is lined with a single layer of epithelial cells that are renewed every 2–5 days. The intestinal epithelium comprises crypts, which contain ISCs, and villi, which contain differentiated and specialized intestinal cells. The rapidly cycling ISCs are located at the basement membranes of the crypts, and they maintain continuous epithelial regeneration and epithelial homeostasis under intact circumstances (Sailaja et al., 2016).

Epithelial intestinal cells can be rapidly activated, and thus, ISCs require extraordinarily precise control. The ability of ISCs to reenter the cell cycle upon request critically depends on their ability to maintain a quiescent state. In ISCs, an elevated basal level of autophagy maintains cellular function, whereas the blockade of autophagy in the intestinal tract leads to the premature death of ISCs. Autophagy-deficient ISCs exhibit increased DNA damage and cell cycle arrest (Trentesaux et al., 2020). Knocking out *Atg5* in ISCs causes ROS accumulation, which in turn decreases the number of ISCs in the pool (Asano et al., 2017). Another study showed that mitophagy is activated by the innate immune receptor NOD2 to reduce mitochondrial ROS levels and thus protect ISCs (Levy et al., 2020). Therefore, autophagy is considered as an essential factor in sustained cell proliferation and the preservation of the stem cell pool.

### Disease-Relevant Mechanism of Autophagy in ISCs

Autophagy plays a dual role in ISC-related diseases. Slit2 is a secreted glycoprotein that belongs to the Slit family. It binds to the roundabout receptor Robo1, which activates the Slit2/Robo1 pathway. In ulcerative colitis, the Slit2/Robo1 pathway regulates ISC proliferation to attenuate inflammation in the colon by activating autophagy (Xie et al., 2020).

Similarly, in the early stage of tumorigenesis, tumor suppressive autophagy inhibits the excessive proliferation and malignant transformation of ISCs by degrading epidermal growth factor receptor (EGFR) (Zhang et al., 2019a). Autonomous autophagy deficiency leads to the induction of intestinal hyperplasia and colon cancer (Zhang et al., 2019b). However, in the later stage of tumorigenesis, autophagy is hijacked by CSCs to provide energy and nutrients for themselves. Prox1 is a transcription factor involved in tumor progression (Elsir et al., 2012), and it is not expressed in normal ISCs. Under pathological conditions, Prox1 promotes ISC survival through autophagy activation, thus facilitating tumor growth (Wiener et al., 2014).

### Physiological Role of Autophagy in iPSCs

In 2006, the research team of Shinya Yamanaka was the first to induce and designate “induced pluripotent stem cells.” These cells are similar to embryonic stem cells with respect to morphology; gene expression profile; and many crucial functions, including cell multiplication, embryogenesis, teratogenesis, and the capacity to differentiate and form chimeras (Takahashi and Yamanaka, 2006).

The stemness of iPSCs is maintained by a high level of autophagic flux, which also prevents genomic defects and ROS-induced injury. Autophagy clears the mitochondria during iPSC reprogramming, during which a necessary metabolic transformation from mitochondrial oxidative phosphorylation to glycolysis occurs (Xu et al., 2013). In other words, autophagy protects the self-renewal and differentiation capacities of iPSCs. In a previous study, the autophagy inhibitor 3-MA was used to evaluate the induction and protective effects of autophagy in iPSCs. Notably, the authors determined that the loss of autophagy leads to a near-complete block of iPSC proliferation (Ma et al., 2015).

### Disease-Relevant Mechanism of Autophagy Concerning iPSCs

Many disease and drug mechanisms have been studied in cellular and animal models. However, there are differences between animal models of disease and human disease. Due to their pluripotency, iPSCs have the ability to generate different types of tissues. Thus, iPSCs derived from patients have been extensively used to establish models of diseases, such as neurodegenerative diseases (Amin et al., 2019; Seranova et al., 2020), providing a new and powerful model for disease research.

For example, autophagy impairment has been shown to be a pathological characteristic of AD that is caused by presenilin 1 deficiency (Chong et al., 2018). These results were previously confirmed by Martin-Maestro using a patient-derived iPSC model of AD (Martin-Maestro et al., 2017). Moreover, iPSCs from AD patients can be used as an experimental model to explore the mechanism of drugs used for AD treatment. Dantrolene has been approved by the Food and Drug Administration for the treatment of malignant hyperthermia, but it has also been shown to help prevent memory decline in animal models of AD. In iPSCs from patients with AD, dantrolene restores intracellular Ca^2+^ homeostasis and physiological autophagy, thus attenuating impaired neurogenesis and synaptogenesis (Wang et al., 2020). In another pharmacological study, iPSC-derived NSCs were used to explore the effect of bexarotene, an autophagy-activation drug, on AD (Martin-Maestro et al., 2019). Taken together, these results suggest that iPSCs derived from patients may be an ideal model for research on related diseases.

### Physiological Role of Autophagy in CSCs

Autophagy, as a ubiquitous survival pathway in cells, is closely linked with cancer, in which it plays a dual role as either a tumor suppressor or a tumor promoter (Yun et al., 2021). In the early stage of tumorigenesis, autophagy maintains cellular homeostasis through protein and organelle quality control mechanisms (Li et al., 2020). Autophagy sustains genomic stability and inhibits the occurrence of inflammation by degraded oncogenic proteins, thus hindering tumor initiation, development, and metastasis (Zhong et al., 2016; Chao et al., 2021). The tumor-suppressive function of autophagy can also be evidenced by the fact that knocking down *Atg7* promotes cell proliferation in non-small cell lung cancer (NSCLC) cells (Cao et al., 2020). However, at the later stage of tumorigenesis, autophagy helps cancer cells survive and adapt to unfavorable microenvironments, such as hypoxia and nutrient deprivation (Xue et al., 2016; Guo et al., 2018). Moreover, autophagy inhibition reduces drug resistance in gastric cancer (Xin et al., 2019), multiple myeloma (Zhang et al., 2020), colorectal carcinoma (Wang and Gu, 2018), and NCSLC (Lotsberg et al., 2020). Accordingly, autophagy plays an important role in tumorigenesis. It is reasonable to speculate that autophagy is necessary for CSCs to continuously generate new cancer cells.

Cancer stem cells are functionally similar to stem cells, but they can drive tumorigenesis. Tumor cells can form a cellular hierarchy similar to that in normal tissue, in which CSCs remain at the peak and control the occurrence, malignant transformation, drug resistance, and recurrence of tumors. Compared with normal stem cells, CSCs exhibit dysregulated migration and invasion abilities, as well as an abnormal tolerance to pharmacologic and immune factors (Smith and Macleod, 2019). There is increasing evidence suggesting that CSC maintenance and differentiation rely on autophagy (Camuzard et al., 2020; Yao et al., 2020; Wang et al., 2021).

Cancer stem cells reside in the niche, which is also a part of the tumor microenvironment. However, the niche has anatomically distinct regions within the tumor microenvironment that maintain the key properties of CSCs and protect them from the immune system (Plaks et al., 2015). The microenvironment has been shown to provide some necessary factors for the stability of stem cell niches so that the stem cells retain their properties. Autophagy is a process that is necessary in the tumor microenvironment, and it may modulate the interaction between tumor cells and components of both the innate and adaptive immune systems and supply nutrients or other factors to regulate the growth of tumor cells (Levy et al., 2017). For example, autophagy promotes the release of damage-associated molecular patterns and ATP from dying tumor cells, thus recruiting CD8^+^ cytotoxic T lymphocytes that synergize with conventional therapeutics to eliminate cancers (Michaud et al., 2011).

**FIGURE 2:**
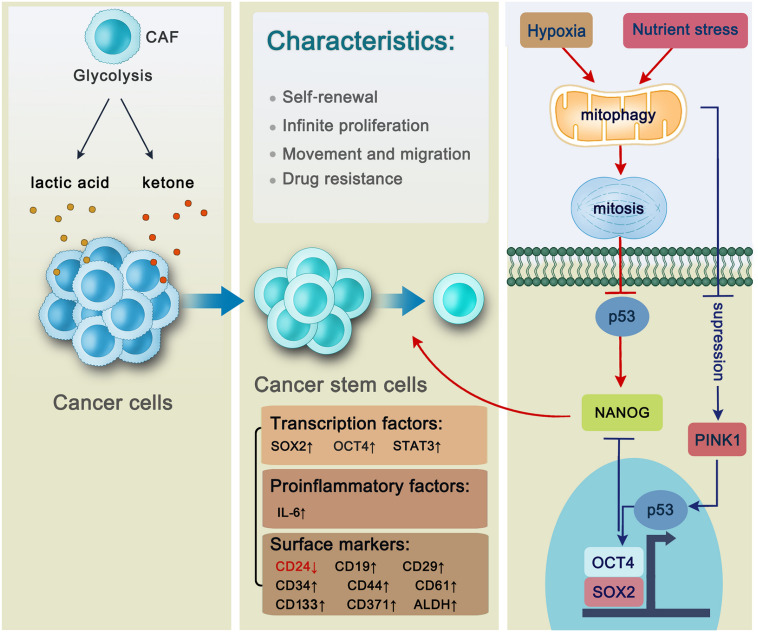
The general characteristics of cancer stem cells and the regulation of mitophagy in hepatic cancer stem cells. Cancer cells benefit from cancer-associated fibroblasts whose products including anaerobic glycolysis lactic acid and ketone are utilized by cancer cells to generate new cancer stem cells. Cancer stem cells are characterized by self-renewal, infinite proliferation, movement and migration and drug resistance with a series of intracellular factors and surface markers changing. Transcription factors including SOX2, OCT4, and STAT3 are activated through autophagy-related pathways to be involved in the self-renewal of hepatic CSC. Autophagy promotes the maintenance of CD44^+^/CD24^*low/*–^ phenotype in the MDA-MB-468 breast cancer cell lines via proinflammatory factor IL-6 secretion. CD133 is a stem cell marker located on both liver cancer stem cells and pancreatic cancer stem cells. In AML, LSCs express surface markers including CD371. In B-ALL, CD34, and CD19 are located on the surface of LSCs. There are three kinds of cancer stem cells in the breast cancer, whose surface markers are characterized by CD44^+^/CD24^*low/*–^, ALDH^+^, and CD29^*hi*^CD61^+^, respectively. Mitophagy plays an essential role in hepatic CSC maintenance. Specifically, mitophagy will be activated under the unfavorable condition such as hypoxia or nutrient stress followed by p53 inhibition, thus promoting NANOG expression which can facilitate stemness of liver CSCs. However, if mitophagy is suppressed, PINK1 will be activated to phosphorylate p53 at S392, making it possible for p53 to translocate into the nucleus. Then p53 binds to the NANOG promoter, leading to the failure of OCT4 and SOX2 to reach to their binding sites on the NANOG promoter, thus suppressing NANOG expression.

However, another study reported that autophagy is induced in certain other non-tumor cells in the tumor microenvironment in a manner that further promotes tumor cell growth and progression (Sousa et al., 2016). To adapt to conditions of hypoxia, cancer cells prefer to generate energy through glycolysis, a process that generates pyruvate and lactate. This is termed the “Warburg effect” (also known as aerobic glycolysis) (Gentric and Mechta-Grigoriou, 2021). However, a novel metabolite pathway known as the “reverse Warburg effect” is also used by cancer cells. Specifically, epithelial cancer cells induce oxidative stress in cancer-associated fibroblasts (CAFs), followed by mitophagy activation in CAFs. The CAFs are forced to undergo aerobic glycolysis, whose energy-rich metabolites pyruvate and lactate are utilized by cancer cells to undergo the TCA cycle for abundant energy production (Martinez-Outschoorn et al., 2011), thereby promoting the generation of new CSCs (Pavlides et al., 2009; Figure 2).

### Disease-Relevant Mechanism of Autophagy in CSCs

During early tumorigenesis, autophagy maintains intracellular homeostasis by removing damaged mitochondria, peroxisomes, and other cytotoxic substances from normal cells, thus inhibiting the activation of oncogenes and preventing further tumorigenesis. In malignantly transformed cancer cells, however, autophagy recycles cellular components to provide nutrients that are essential for survival, thereby promoting cell proliferation, invasion, and metastasis. In this section, we will mainly focus on the role of autophagy in hepatic and breast CSCs, and discuss the intersection between autophagy and drug resistance in other types of CSCs from cancers including epithelial ovarian cancer, bladder cancer, pancreatic cancer, glioblastoma, and gastric cancer.

In hepatoma carcinoma, chloroquine, an autophagy inhibitor, reduces the formation of autophagic lysosomes, blocks pSTAT3, and inhibits the self-renewal of hepatic CSCs (Li et al., 2017). Moreover, studies have shown that mitophagy is also involved in cancer progression. Specifically, when mitophagy is impaired in hepatic CSCs, the tumor suppressor p53 is phosphorylated at S392 by PINK1, a kinase associated with mitophagy, in the mitochondria. p53 then translocates to the nucleus and binds to the *NANOG* promoter, causing a failure of OCT4, SOX2 transcription factors to activate expression of NANOG which is a pivotal transcription factor in maintenance of hepatic CSCs (Liu K. et al., 2017). Conversely, mitophagy can be enhanced under conditions such as hypoxia and starvation. After p53 is inhibited by mitophagy, it localizes in the mitochondria and is degraded in a mitophagy-dependent manner, after which it cannot affect NANOG expression, thereby promoting the self-renewal of hepatic CSCs (Lee et al., 2018). These findings suggest that autophagy activates hepatic CSCs and thus facilitates their stemness and self-renewal (Figure 2).

CD133 is a biomarker of stem-like cells, and its expression has been demonstrated in liver CSCs (Suetsugi et al., 2006). In low-glucose medium, CD133 has been shown to promote the uptake of glucose and the generation of sufficient energy for the survival of Huh-7 cells (human hepatoma cells), in which autophagy is activated (Chen et al., 2013a). A monoclonal anti-CD133 antibody (CD133mAb) has also been shown to promote the death of hepatoma cells expressing CD133 on their surface, by repressing autophagy (Chen et al., 2013b). Notably, the tumor-suppressive effect of CD133mAb on liver CSCs is more effective under conditions of nutrient deprivation, such as in low-glucose medium. Therefore, combined therapy with a hypoglycemic agent, which simultaneously decreases glucose concentration in the tumor microenvironment, increases the sensitivity of CSCs to CD133mAb.

In breast cancer, tumor growth and metastasis require blood vessels, whereas tumor stem cells differentiate into endothelial cells to promote the formation of new blood vessels. The levels of autophagy-related factors have been shown to be elevated during the differentiation of breast cancer stem cells (BCSCs), and the use of autophagy blockers or the conditional knockout of *Atg5* inhibits the differentiation process (Yao et al., 2020). In the process of tumor cell metastasis, there is excessive blood supply, resulting in hypoxia, a lack of nutrients, and other unfavorable conditions. Autophagy helps BCSCs combat hypoxia and perform protein catabolism to supply BCSCs under conditions of starvation (Espina et al., 2010). When antiangiogenic drugs are used to treat breast cancer, instead of inhibiting the development of the cancer, they have been found to promote metastasis by generating intratumoral hypoxia (Conley et al., 2012). As we discussed above, the increase in BCSC metastasis may attribute to the hypoxic microenvironment formed by antiangiogenic drugs, under which autophagy is activated to provide sufficient energy for BCSC metastasis and tumor development.

Research on the relationship between autophagy and BCSCs must be designed specifically based on different types of BCSCs, because the autophagic process differs in different cell types. Based on surface markers, there are three distinct CSC types, namely CD44^+^/CD24^*low/*–^, ALDH^+^, and CD29^*hi*^CD61^+^ phenotypes (Ricardo et al., 2011; Yeo et al., 2016). Although all BCSCs have the potential for tumor initiation, autophagy promotes their maintenance via different pathways. For example, autophagy promotes the maintenance of CD44^+^/CD24^*low/*–^ MDA-MB-468 breast cancer cells via IL-6 secretion. However, autophagy does not promote the survival of CD44^+^/CD24^*low/*–^ MCF-7 CSCs (Maycotte et al., 2015). Autophagy has also been implicated in the tumor-initiating potential of distinct BCSC subsets through different pathways (Yeo et al., 2016). Autophagy deficiency induced by FIP200 depletion causes a decrease in the number of CD29^*hi*^CD61^+^ BCSCs and their potential to initiate tumors by inhibiting the TGF-β/Smad pathway. However, the inhibition of autophagy by *FIP200* knockout reduces the number of ALDH^+^ BCSCs as well as their tumor-initiating potential. In summary, there is a need for a comprehensive consideration of how to utilize autophagy against these three distinct types of BCSCs.

Triple-negative breast cancer (TNBC) is an important form of breast cancer. The drug resistance and recurrence of TNBC are mainly attributed to the ability of CSCs to generate an unlimited number of new cancer cells, during which autophagy plays an important role. Data from patient-derived xenograft models indicate that autophagy is activated in chemo-resistant xenografts, as evidenced by higher levels of autophagy markers (*BECN1* mRNA) than those detected in chemo-sensitive models (Bousquet et al., 2017). This study implies that autophagy inhibition may be effective for cancer therapy. Chloroquine (an autophagy inhibitor) has been used to target CSCs to inhibit autophagy, which causes damage to the mitochondrial structure, increased oxidative stress levels, and changes in the degree of DNA methylation, thereby reducing the metastatic potential of TNBC (Liang D.H. et al., 2016). Furthermore, the combination of chloroquine and standard chemotherapy exerts favorable effects. For example, chloroquine sensitizes TNBC cells to paclitaxel by inhibiting autophagy and decreasing the population of CD44^+^/CD24^–/low^ CSCs by blocking the Janus-activated kinase (JAK) 2/STAT3 pathway (Choi et al., 2014). The anti-tumor effect of pterostilbene, a natural demethylated analog of resveratrol from blueberries, potentiates the effect of the autophagy inhibitor 3-MA (Chen et al., 2014). Accordingly, autophagy inhibition is a promising target for TNBC treatment and a combined therapy is worth consideration.

Links have also been identified between autophagy and other types of CSCs, including those in epithelial ovarian cancer, bladder cancer, pancreatic cancer, glioblastoma, and gastric cancer. In view of the role of autophagy in the resistance of tumors to chemotherapy, the tumor suppressor gene *BRCA1* has been shown to render epithelial ovarian CSCs resistant to cisplatin (a chemotherapeutic drug) by regulating autophagy (Li D. et al., 2016). Autophagy inhibitors may attenuate the drug resistance of epithelial ovarian CSCs mediated by BRCA1 (You et al., 2019). A recent study has also demonstrated that the autophagy inhibitor chloroquine in combination with cisplatin decreases the drug resistance of epithelial cancer cells (Hwang et al., 2020). These results indicate that autophagy may be a promising target for overcoming drug resistance in epithelial ovarian cancer.

Similarly, autophagy is also linked to the drug resistance of bladder cancer cells. Greater autophagic flux has been observed in bladder CSCs (Ojha et al., 2014). Atg7 promotes the maintenance of bladder CSCs by stabilizing CD44, which serves as a classical marker of stem cells and also favors sphere formation, invasion, and lung metastasis (Zhu et al., 2019). JAK2 expression activates autophagy in CSCs, leading to increased expression levels of genes related to drug resistance in CSCs (Ojha et al., 2016). Accordingly, autophagy inhibition may be an effective therapeutic strategy. Icaritin, a flavonol glycoside, delays the progression of bladder cancer by inhibiting autophagy (Pan et al., 2016). Furthermore, autophagy inhibition can render CSCs more sensitive to the chemotherapeutic drugs, cisplatin (Ojha et al., 2014) and taxol (Ma et al., 2021).

In a hypoxic microenvironment, increased levels of HIF-1α induce autophagy to promote the transformation from non-stem pancreatic cancer cells to CD133^+^ pancreatic CSCs (Zhu et al., 2013). Additionally, HIF-1α-induced autophagy has been shown to promote the epithelial-to-mesenchymal transition of pancreatic CSCs, resulting in increased tumor aggressiveness (Zhu et al., 2014). Rausch reported that a balanced autophagy flux, with neither hyperactivation nor over-inhibition, maintains pancreatic CSC survival (Rausch et al., 2012). The stem cell-like characteristics of pancreatic cancer cells are diminished following the inhibition of nutrient-deprivation autophagy factor-1, an important factor that regulates autophagy and oxidative stress (Qin et al., 2020). Autophagy is also involved in drug resistance, as exemplified by the negative regulation of pancreatic CSC stemness by mitophagy impairment as a result of ISGylated protein depletion (Alcala et al., 2020). The addition of chloroquine inhibits pancreatic CSC activity and increases the anti-tumor effect of gemcitabine (Yang et al., 2015).

The autophagy-related factor DRAM1 promotes to the localization of p62, an essential regulator of autophagy, to autophagosome. p62 has a short LC3-interacting region, which can promote binding with LC3, which results in the selective degradation of p62 through autophagy (Li et al., 2020). Defective autophagy causes p62 accumulation, and therefore, p62 is a negative marker of autophagy activation. DRAM1 and p62 regulate the migration and invasion of glioma stem cells (GSCs) and autophagy-mediated cell degradation (Galavotti et al., 2013). GSCs and other glioblastoma cells survive and promote tumor growth through the induction of autophagy and the inhibition of apoptosis under a hypoxic microenvironment (Chakrabarti et al., 2016). A demethoxycurcumin analog is used to inhibit the growth of glioblastomas via the activation of apoptosis, but it also induces autophagy, which protects GSCs from apoptosis (Shi et al., 2020). Therefore, the combination therapy of demethoxycurcumin and an autophagy inhibitor may be more effective than monotherapy for cancer treatment. The PI3K/AKT pathway is considered to be involved in the maintenance of GSCs. Treatment with chloroquine, while concomitantly inhibiting the PI3K/AKT pathway, significantly inhibits glioblastoma progression (Graham et al., 2014). GSCs are resistant to temozolomide, an oral anti-tumor drug. A low concentration of temozolomide can promote the growth of GSCs. A recent study suggested that GSC-derived PD-L1-containing exosomes activate AMPK/ULK1-mediated autophagy, thus increasing drug-resistance in glioblastoma (Zheng et al., 2021). Accordingly, autophagy inactivation increases the sensitivity of GSCs to drugs (Buccarelli et al., 2018).

The level of autophagy is significantly higher in CD44^+^ CD54^+^ gastric CSCs than in non-CSCs. There is considerable evidence to suggest that Notch signaling regulates gastric CSC resistance. Combined treatment with chloroquine (an autophagy inhibitor) and 5-fluorouracil (a chemotherapeutic agent) inhibits CSC activity, increases the expression levels of Notch1 protein, and increases the sensitivity of cells to drugs (Li et al., 2018). In *Helicobacter pylori*-infected gastric cancer, levels of the autophagy marker LC3 increase concomitantly with the upregulation of CD44 (Courtois et al., 2021), a classical marker of stem cells, indicating that autophagy plays a vital role in gastric CSCs. However, incomplete autophagy also exists in gastric CSCs to protect *H. pylori* from contact with antibiotics.

## Conclusion

The highly conserved process of autophagy works as a “cleaner” to degrade damaged proteins and impaired organelles in cells. This process contributes to the homeostasis of adult stem cells in terms of their quiescence, self-renewal, and differentiation. Dysfunctional autophagy in adult stem cells leads to the progression of diseases, especially various types of cancers. Autophagy plays different roles in different stages of tumorigenesis, such as initiation, development, and metastasis, either inhibiting tumor growth or promoting tumor development. In the earlier stages of tumor progression, autophagy regulates cellular homeostasis by eliminating damaged mitochondria and oncogenic proteins, thus inhibiting tumor initiation and delaying tumor growth. However, in the later stages of tumorigenesis, autophagy is a survival mechanism for cancer cells, allowing them to acquire energy and materials through autophagic degradation, which helps them to adapt to unfavorable environments, thereby promoting tumor progression, metastasis, and invasion. Although autophagy inhibition has been shown to be a promising strategy for cancer treatment (Yang et al., 2015; Pan et al., 2016; Buccarelli et al., 2018; Bie et al., 2021), there are some studies indicating that autophagy activation can also be utilized in cancer treatment. For example, autophagy activation, not inhibition, increases the sensitivity of GSCs to drugs (Hung et al., 2020). Another study showed that the co-suppression of mTORC1, histone deacetylase, and estrogen receptor 1 decreases the number of CSCs via autophagy activation in TNBC (Sulaiman et al., 2018). These results are reasonable based on the dual role of autophagy in tumorigenesis. Consequently, before utilizing autophagy as a target for cancer treatment, the tumorigenesis stage, the context, microenvironment stress levels, and nutrient availability need to be taken into consideration.

A study published in 2018 showed that autophagy suppression increased the sensitivity of GSCs to temozolomide, which is used for glioblastoma treatment through the activation of ferroptosis (an iron-dependent form of programmed cell death) (Buccarelli et al., 2018). This suggests that autophagy suppression may affect other intracellular events, such as ferroptosis and apoptosis, indicating essential roles for autophagy during these cellular processes (Jang et al., 2017, 2020). In addition, in the transplantation of bone-derived MSCs for the treatment of myocardial infarction and myocardial and cerebral I/R injury, targeting autophagy may significantly improve therapeutic efficacy. Therefore, targeting autophagy is a promising strategy not only for cancer but also for transplantation treatment.

In summary, our review has comprehensively demonstrated the essential roles of autophagy in CSC regulation and CSC-related diseases, suggesting that autophagy is a promising target for clinical therapy.

## Author Contributions

NW and DW provided the idea, designed the structure of the manuscript and figures, and revised the manuscript. SC, WW, and H-YT collected materials, wrote and revised the manuscript, and drew the figures and revised them. YL collected materials and provided many valuable comments. ZL provided useful advice during the process of revision. YQ collected materials and revised the manuscript. YL, ZL, and YQ provided useful advice during the drawing process of figures. All authors contributed to the article and approved the submitted version.

## Conflict of Interest

The authors declare that the research was conducted in the absence of any commercial or financial relationships that could be construed as a potential conflict of interest.

## Publisher’s Note

All claims expressed in this article are solely those of the authors and do not necessarily represent those of their affiliated organizations, or those of the publisher, the editors and the reviewers. Any product that may be evaluated in this article, or claim that may be made by its manufacturer, is not guaranteed or endorsed by the publisher.

## Abbreviations

ESCs: embryonic stem cells
ATG: autophagy-related genes
LC3: microtubule-associated protein 1 light chain 3
HSCs: hematopoietic stem cells
NSCs: neural stem cells
MSCs: mesenchymal stem cells
ISCs: intestinal stem cells
iPSCs: induced pluripotent stem cells
CSCs: cancer stem cells
LSCs: leukemia stem cells
AML: acute myeloid leukemia
CML: chronic myeloid leukemia
B-ALL: B-cell acute lymphoblastic leukemia
ZIKV: Zika virus
AD: Alzheimer’s disease
BMSCs: bone marrow mesenchymal stem cells
CAFs: cancer associated fibroblasts
TNBC: triple negative breast cancer
GSCs: glioma stem cells
ROS: reactive oxygen species.
